# Systemic transcriptome comparison between early‐ And late‐onset pre‐eclampsia shows distinct pathology and novel biomarkers

**DOI:** 10.1111/cpr.12968

**Published:** 2020-12-17

**Authors:** Fang Guo, Bao Zhang, Hao Yang, Yixi Fu, Yaning Wang, Jianming Huang, Mi Cheng, Xiaobo Li, Zhuojian Shen, Li Li, Ping He, Andy Peng Xiang, Shuaiyu Wang, Hongbo Zhang

**Affiliations:** ^1^ Department of Obstetrics First Affiliated Hospital of Jinan University Guangzhou China; ^2^ Department of Obstetrics, Guangzhou Women and Children's Medical Center Guangzhou Medical University Guangzhou China; ^3^ Key Laboratory for Stem Cells and Tissue Engineering Ministry of Education Sun Yat‐sen University Guangzhou China; ^4^ The Department of Histology and Embryology Zhongshan School of Medicine, Sun Yat‐sen University Guangzhou China; ^5^ Core Facilities for Medical Science Sun Yat‐sen University Guangzhou China; ^6^ Department of Thoracic Surgery, Guangdong Provincial Key Laboratory of Malignant Tumor Epigenetics and Gene Regulation Sun Yat‐sen Memorial Hospital of Sun Yat‐sen University Guangzhou China

**Keywords:** Biomarkers, Maternal blood, Placenta, Pre^‐^eclampsia, Single‐cell RNA sequencing

## Abstract

**Objectives:**

Pre‐eclampsia is a leading cause of morbidity and mortality during pregnancy. Although the two forms of this disorder, early‐ (EOPE) and late‐onset of pre‐eclampsia (LOPE) are different, the underlying pathology remains elusive. We aim to unravel the difference and to identify novel biomarkers for EOPE and LOPE.

**Materials and Methods:**

A complete comparison of both placental and peripheral blood transcriptomes was performed to investigate the pathology of pre‐eclampsia. Single‐cell transcriptomics of the maternal‐fetal interface were integrated to identify novel biomarkers for EOPE and LOPE which were further verified at protein or mRNA level in patients.

**Results:**

We found that the transcriptomes of placentae from EOPE, but not LOPE, were significantly different from their respective controls. Conversely, the transcriptomes of peripheral blood from LOPE were more different from their controls than EOPE. Importantly, we identified that several classical biomarkers of pre‐eclampsia were expressed specifically in extravillous trophoblast and syncytiotrophoblast and only upregulated in EOPE, suggesting they should not be applied to all pre‐eclampsia patients in general. We further identified novel biomarkers for EOPE and LOPE from differentially expressed genes (DEGs) of placental and peripheral blood, respectively. The new biomarkers EBI3, IGF2, ORMDL3, GATA2 and KIR2DL4 were experimentally verified with patient blood samples.

**Conclusion:**

Our data demonstrate distinct pathology of EOPE and LOPE, and uncover new biomarkers that can be applied in diagnosis for pre‐eclampsia.

## Graphical abstract

The schematic diagram shows the distinct pathogenesis between early‐ (EOPE) and late‐onset pre‐eclampsia (LOPE). The difference between EOPE and LOPE is demonstrated at several layers, such as risk factors, signalling pathways and disease affected/causing tissues. Based on divergence of EOPE and LOPE pathogenesis, specific biomarkers can be identified and used to distinguish both diseases by testing placental secreted proteins or maternal blood cell transcripts.

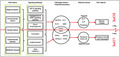

## 1 INTRODUCTION

Pre^‐^eclampsia, one of the most severe hypertensive disorders of pregnancy, threatens 4%‐5% of gravidas in the world and is a leading cause of maternal and neonatal morbidity and mortality.^1^, ^2^ As a disease with heterogeneous aetiology and diverse clinical symptoms, pre‐eclampsia is defined as the presence of new‐onset hypertension and proteinuria or end‐organ damage occurring after 20 weeks of gestation.^3^ Once diagnosed, controlling blood pressure is the mainstay of clinical intervention. However, given that the underlying pathogenesis remains elusive, there is no cure for pre‐eclampsia and delivery of placenta remains the only definitive treatment.

In the past two decades, various mechanisms have been proposed causing pre‐eclampsia, including defective placentation, imbalance in circulating angiogenic factors, placental ischaemia and hypoxia, abnormal immune interaction at the maternal‐foetal interface, and renin‐angiotensin pathway, though none have conclusive evidence in humans.^4^ Among them, elevated antiangiogenic factors, such as fms related receptor tyrosine kinase 1 (FLT1) and endoglin (ENG), have emerged as key pathogenic mediators of maternal pre‐eclampsia.^5^, ^6^ These factors, together with leptin (LEP) and the regulator of insulin‐like growth factor bioavailability pappalysin 2 (PAPPA2), have also provided opportunities for the development of biomarkers for the diagnosis and prediction of pre‐eclampsia.^7^, ^8^, ^9^, ^10^ However, the test inaccuracy of these markers restricts their wide use in clinical practice, triggering harsher clinic trials applied to evaluate the efficiency and sensitivity of diagnosis.^11^ Therefore, the discovery of novel biomarkers through deeper understanding the pathology of diseases is currently an urgent task.

Besides, pre‐eclampsia can be further categorized into early‐ (EOPE, < 34 weeks of gestation) and late‐onset of pre‐eclampsia (LOPE, ≥ 34 weeks of gestation) based on the timing of clinical symptoms present.^12^ The variability of clinical implications, long term outcome and inconsistent response to preventive treatments suggest the pathological discrepancy between EOPE and LOPE. It is well acknowledged that EOPE is associated with abnormal placentation secondary to defective remodelling of the uterine spiral arteries, while LOPE is more likely due to the imbalance between senescence of the placenta and a maternal predisposition to cardiovascular and metabolic diseases.^13^, ^14^, ^15^ Nevertheless, the detail in differences between EOPE and LOPE is still poorly understood.

In the present study, we integrated publicly available resources and performed a systemic transcriptome comparison of both placental and peripheral blood transcriptomes to investigate foetal and maternal differences between women diagnosed with EOPE, LOPE and their controls. Our results demonstrate fundamental pathologic differences between EOPE and LOPE, and reveal novel blood circulating factors or maternal blood transcripts as biomarkers for EOPE and LOPE, respectively. We suggest EOPE and LOPE should be treated as two distinct disease entities with different markers.

## 2 MATERIALS AND METHODS

### 2.1 Bioinformatic analysis of transcriptomic profiles

#### 2.1.1 Data collection, cleaning and pre‐processing

The transcriptomic expression profiling of placentae (GSE74341) and maternal peripheral blood (GSE48424) were collected from previous published data.^16^, ^17^ Both datasets cover the same pregnant stages from 31 to 37 gestational weeks, and the diagnostic criteria and severe symptoms are followed the standard defined by American College of Obstetricians and Gynecologists (ACOG).^3^ Placental and maternal peripheral blood data were cleaned and pre‐processed by using the limma (R package)^18^ with the same pipeline. Detailly, background correction was performed using the ‘normexp’ method; on‐microarray standardization was performed using the ‘loess’ method; inter‐microarray standardization was performed using the ‘quantile’ method.

#### 2.1.2 Sample clustering and identification of differentially expressed genes (DEGs)

The principle component analysis (PCA) was performed by using princomp function of limma with default parameters. The sample clustering was performed by using the text2vec (R package) with all genes as input and with default parameters. DEGs were identified by using eBayes function of limma with default parameters. For placental samples, these genes were identified as DEGs with fold change ≥ 2 and adjusted *P*‐value ≤ 0.05. For maternal peripheral blood samples, DEGs were defined with fold change ≥ 2 and with *P‐*value ≤ 0.01.

#### 2.1.3 Functional enrichment analysis

Functional enrichment analysis of DEGs was performed using the Metascape.^19^ Gene set enrichment analysis (GSEA) was performed using the clusterProfiler (R package).^20^

#### 2.1.4 Co‐expression network construction

Co‐expression network analysis was performed using weighted correlation network analysis (WGCNA, R package).^21^ Genes with variances ranked top 25% were regarded as highly variable genes (HVGs) and selected as input matrix. The co‐expression network was constructed by automatic construction function with the parameter power 10. Co‐expression network was visualized by Cytoscape.^22^

### 2.2 Bioinformatic analysis of single‐cell transcriptomic datasets

#### 2.2.1 Cleaning and pre‐processing single‐cell data

The single‐cell datasets of placental samples were downloaded from ArrayExpress (https://www.ebi.ac.uk/arrayexpress/experiments/E‐MTAB‐6701/) and only those libraries (FCA7196220, FCA7196226, FCA7474064, FCA7474065, FCA7474068 and FCA7511884) created with droplet‐based single‐cell RNA sequencing were included in this study. The raw gene expression matrices of all samples were merged using Python (version 3.6.6) and converted to an Anndata object using the Python package Scanpy (version 1.4.4).^23^ Cells that expressed less than 500 genes and genes detected in less than 3 cells were filtered out. Potential doublet cells were then detected and filtered by applying the Python package scrublet (version 0.2)^24^ for each sample. Next, doublet‐dominated sub‐clusters were checked to ensure low doublet rate in all populations using the method as described.^25^ The gene expression levels were normalized by the total UMI count per cell (1e4) with data being log‐transformed. The interferences arising from cell cycling genes were removed by using the regress_out function of the Scanpy package. Then HVGs in gene expression matrices were identified for further analysis using highly_variabe_genes function of the scanpy package. Finally, the batch effect was eliminated using Python package bbknn (version 1.2.0).^26^

#### 2.2.2 Reducing dimension, clustering and identifying cell‐specific genes

The dimensionality of HVGs were primarily reduced by PCA. The first 40 principal components were further summarized by UMAP dimensionality reduction using the default setting of the umap function of the Scanpy package. Cells were clustered with the Leiden algorithm using the leiden function of the Scanpy package. Cell‐specific gene markers across all cell types were identified with the get_DEG_single function of Python package PLOGS (https://github.com/ZhangHongbo‐Lab/PLOGS) that we own developed, with parameters ratio ≥ 0.5 and *q*‐value ≤ 1e‐30.

#### 2.2.3 Identifying secretory protein‐coding genes and constructing protein‐protein interaction network

The reference list of secretory proteins was downloaded from previous study.^27^ The intersection of the reference list, the DEGs and cell‐specific genes were considered as the differentially expressed‐secretory protein‐coding genes, all of which were applied to GeneCards database (https://www.genecards.org/) for further confirmation. The STRING database (https://string‐db.org) was then searched to fetch genes which interact with the above genes and Cytoscape was used to construct the secretory protein‐protein interaction network.

### 2.3 Women peripheral blood sampling

The maternal peripheral blood was collected from the Guangzhou Women and Children's Medical Center under the licence No. 2020‐028 approved by the medical ethics committee of Zhongshan School of Medical, Sun Yat‐sen University. Women with a singleton pregnancy had normal blood pressure and no history of medical illness or use of medication before pregnancy. The diagnosis of women with pre‐eclampsia and severe symptoms were based on the report of ACOG.^3^ The clinical characteristics of healthy pregnant women and women with pre‐eclampsia are listed in Table 2. Data were presented as mean ± SD. The urinary protein levels of two patients in non‐severe LOPE group were undetectable and thus considered as 0 g in calculation.

Blood samples were collected into EDTA‐Vacutainer tubes (Improve Medical, 101 680 967), placed on ice and centrifuged at 1500 × *g* at 4°C for 5 minutes. The plasma was stored in aliquots at −80°C and blood cells were immediately processed for RNA extraction.

### 2.4 RNA extraction and quantitative PCR (qPCR)

Total peripheral blood cell RNA was extracted with TRIzol (Invitrogen, 15 596 026), and the cDNA was synthesized using the PrimeScript RT Master Mix Kit (TaKaRa, RR036A). The qPCR was carried out using PerfectStart^TM^ Green qPCR SuperMix (TransGen Biotech, AQ601) on a Real‐time PCR Detection System (Roche, LightCycle480 II). *RPL13A* was served as internal control. Primers (5’‐3’) are listed in Table S5.

### 2.5 Enzyme‐linked immunosorbent assay (ELISA)

Plasma biomarker concentrations were measured by commercial ELISA kits for EBI3 (R&D Systems; DY6456‐05), according to the manufacturer's instructions. The lowest detection limit was 62.5 pg/mL.

### 2.6 Statistical analysis

Data were presented as mean ± SEM. Analytical comparisons were performed using the empirical Bayes moderated t‐statistics test (for bioinformatic data) and Student's t‐test (for experimental data). Differences were considered significant with *P < .05, **P < .01 and ***P < .001.

## 3 RESULTS

### 3.1 Comparative transcriptomic profiling of placentae from EOPE and LOPE patients

**FIGURE 1:**
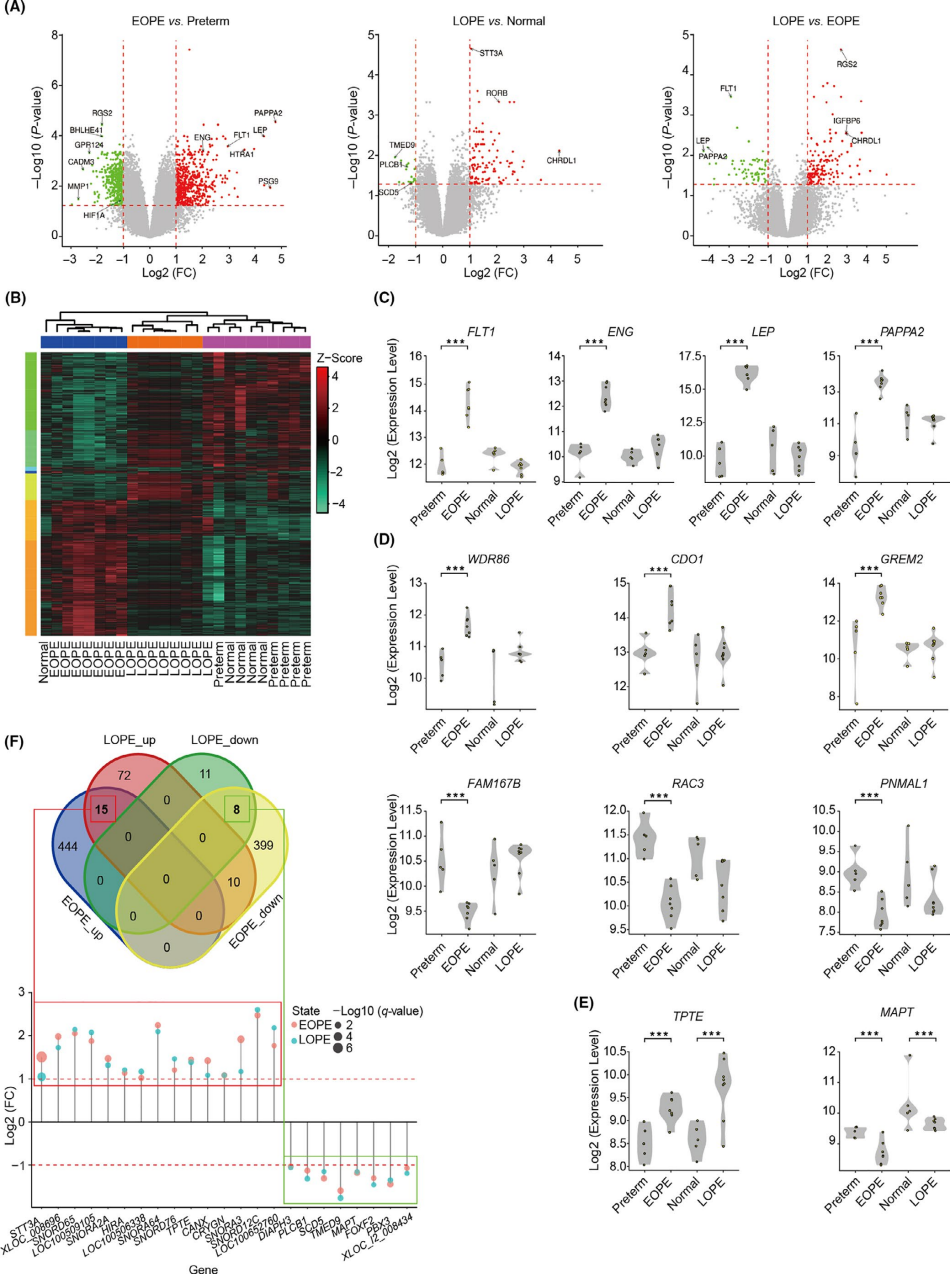
Transcription profiling of placentae with EOPE and LOPE. A, Volcano plots showing DEGs in EOPE (n = 7) *vs*. preterm (n = 5), LOPE (n = 8) *vs* normal (n = 5), and LOPE *vs*. EOPE placentae. The red, green, and grey dots indicate DEGs that considered to be upregulated, downregulated and non‐difference, respectively. Thresholds are indicated with dash lines. B, Heatmap presentation of relative expression and clustering of DEGs in EOPE, LOPE and corresponding controls. C‐E, Violin plots showing expression levels of known pathogenic factors (C), newly identified DEGs in EOPE (D) and novel common DEGs in both EOPE and LOPE (E) compared to their corresponding controls. Data were compared by the empirical Bayes moderated t‐statistics test. *P < .05, **P < .01, ***P < .001. F, Visualization of 15 upregulated and 8 downregulated DEGs in both EOPE and LOPE. DEGs, differentially expressed genes; EOPE, early‐onset of pre‐eclampsia; EOPE_up, the upregulated DEGs in EOPE; EOPE_down, the downregulated DEGs in EOPE; FC, fold change; LOPE, late‐onset of pre‐eclampsia; LOPE_up, the upregulated DEGs in LOPE and LOPE_down, the downregulated DEGs in LOPE

Given that pre‐eclampsia might be originated from the dysfunction of placentae, we first compared the placental gene expression profiles between different forms of pre‐eclampsia and their corresponding controls by deeply re‐analysing publicly available dataset.^16^ DEGs were identified after data quality control and normalization (Figure S1A‐B and Table S1). We found that the number of DEGs between EOPE and its control was much more than LOPE (Figure 1A), which is consistent with the idea that EOPE is a placental disease. It is worthy of note that a large number of DEGs were detected when comparing EOPE to LOPE gene expression profiles (Figure 1A, right panel), suggesting the differential placental pathology between EOPE and LOPE. The DEGs clustering of two forms of pre‐eclampsia and their controls also showed significant divergence between EOPE and the other three groups (Figure 1B). PCA and sample correlation analysis further confirmed that the gene expression in LOPE was closer similar to the normal pregnant placentae (Figure S2A‐C). Importantly, the expression levels of several known pathogenic factors and diagnostic markers for pre‐eclampsia, such as *FLT1*, *ENG*, *LEP*, and *PAPPA2*, were much higher in EOPE placentae compared to preterm controls, which were not observed in LOPE, indicating that their roles in the pathology and diagnosis of pre‐eclampsia might be restricted to EOPE (Figure 1C).

Our data also captured a large number of novel DEGs, and some of them were specifically upregulated (such as *WDR86*, *CDO1*, *GREM2*) or downregulated (such as *FAM167B*, *RAC3*, *PNMAL1*) in EOPE, while a few (such as *TPTE* and *MAPT*) were coordinately changed in both forms of diseases (Figure 1D‐E). We then searched for the common upregulated or downregulated DEGs between EOPE and LOPE, and identified only 15 and 8 genes that were coordinately changed among 843 and 106 DEGs from EOPE and LOPE, respectively (Figure 1F). Closer examination of these 23 genes found the majority were non‐coding RNAs with unknown functions (Figure 1F).

To explore the different mechanisms of placental dysfunction between EOPE and LOPE, we investigated the biological processes and signalling pathways underlying DEGs in EOPE and LOPE. Functional enrichment analysis showed that there were a few pathways, such as biological processes related to hormone transport, upregulated in both EOPE and LOPE, while with no consistent downregulated pathway enriched (Figure S3A‐C). Intriguingly, plenty of pathways including ‘HIF1 TF PATHWAY’, ‘cell surface interactions at the vascular wall’, ‘blood vessel development’ and ‘placenta development’ were specifically enriched in EOPE but not LOPE, confirming that the dysfunction of placenta is specifically involved in EOPE (Figure S3B and D). Meanwhile, some of the key biological processes provided new insights for the understanding of pre‐eclampsia. For example, the low expression levels of basement membrane proteins (eg laminin) have been implicated with pre‐eclampsia, but its pathogenic role is enigmatic.^28^ We found that ‘basement membrane assembly’ was solely enriched in downregulated DEGs in EOPE (Figure S3D), indicating that dysfunction of laminin might disturb basement membrane assembly and thus triggers EOPE. Indeed, the expression levels of laminin subunit alpha 2 (*LAMA2)*, laminin subunit beta 1 (*LAMB1)*, laminin subunit beta 3 (*LAMB3)* and laminin subunit gamma 3 (*LAMC3)* were decreased in EOPE placentae (Table S1).

### 3.2 Single‐cell expression profiling of DEGs identified in placentae from EOPE and LOPE patients

**FIGURE 2:**
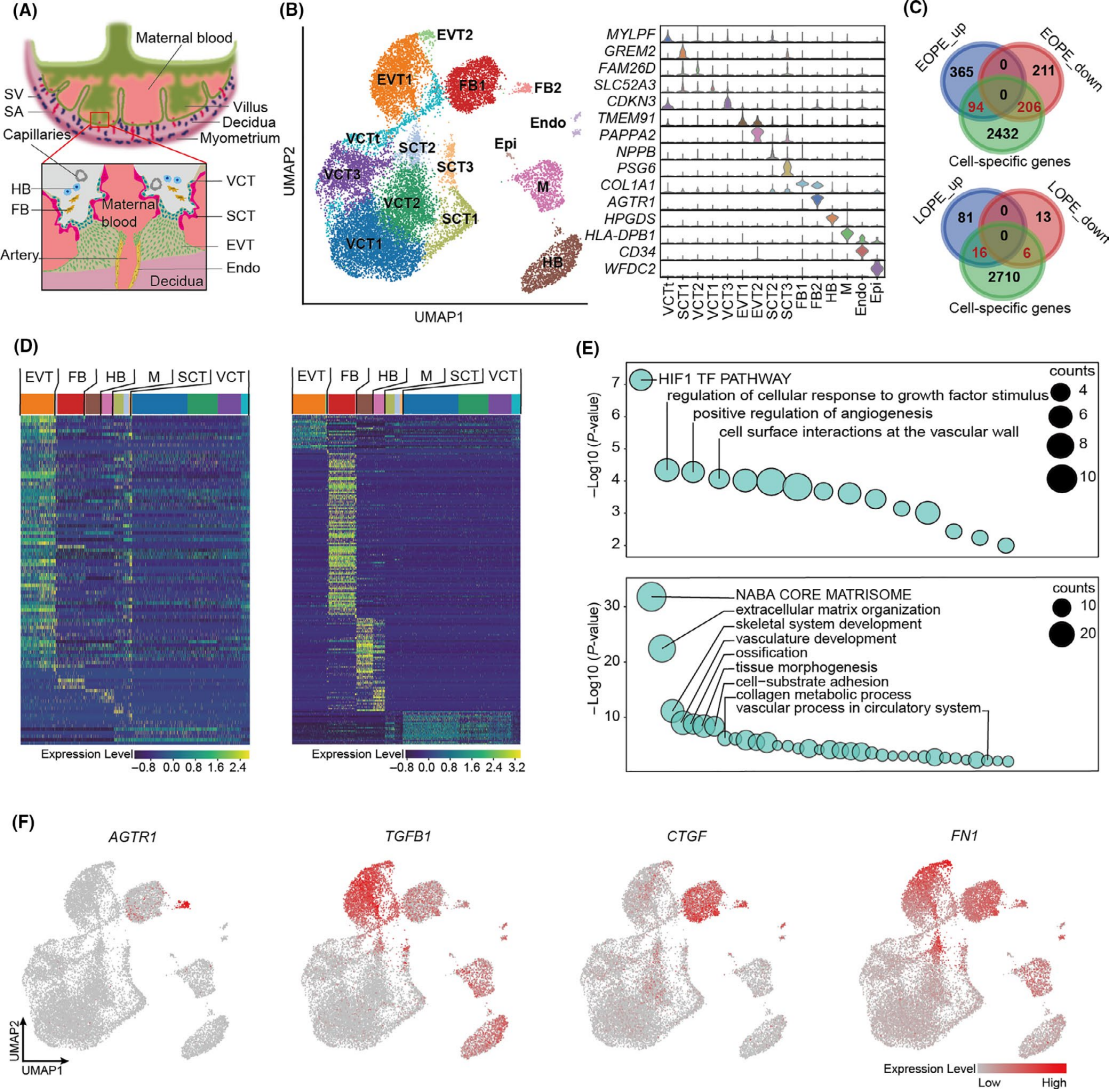
Single‐cell resolution analysis of expression pattern of DEGs in maternal‐foetal interface. A, Diagram illustrating maternal‐foetal interface in normal pregnancy. B, UMAP plot showing all cell clusters and their annotation in the atlas (left panel) based on the marker genes (right panel). C, Venn plots showing the overlap of genes between marker genes and DEGs in EOPE (upper panel) and LOPE (bottom panel). D, Heatmap presenting relative expression of cell‐type‐specific DEGs (upregulated, left panel; downregulated, right panel) in placental cell clusters. E, The enriched GO terms for cell‐type‐specific DEGs in EOPE (upregulated DEGs derived from EVT in top panel, downregulated DEGs derived from FB in bottom panel). F, UMAP plots showing the expression levels of *AGTR1*, *TGFB1*, *FN1* and *CTGF* in the single‐cell atlas of maternal‐foetal interface. Endo, endothelial cell; EOPE_up, the upregulated DEGs in EOPE; EOPE_down, the downregulated DEGs in EOPE; Epi, epithelial glandular cell; EVT, extravillous trophoblast; FB, fibroblast; HB, Hofbauer cell; LOPE_up, the upregulated DEGs in LOPE; LOPE_down, the downregulated DEGs in LOPE; M, macrophage; SA, spiral artery; SCT, syncytiotrophoblast; SV, spiral vein; VCT, villous cytotrophoblast and VCTt, villous cytotrophoblast transient

During normal placental implantation, placental extravillous trophoblast cells (EVT) invade deeply into endometrium to the level of the myometrium, which leads to the remodelling of uterine spiral arteries at the maternal‐foetal interface to provide nutrition to the foetus (Figure 2A).^29^, ^30^ It is known that dysfunction of trophoblast invasion causes the incomplete remodelling of the spiral artery (ie defective placentation), which in turn leads to the hypoxia and oxidative stress at the placenta to induce pre‐eclamptic symptoms.^4^ Many cell types from both placenta and endometrium are involved in the process, however, the culpable cells for pre‐eclampsia have yet to be elucidated (Figure 2A).^31^, ^32^, ^33^ A recently built single‐cell atlas of maternal‐foetal interface provided us opportunity to determine the cell expression specificity of DEGs we identified in EOPE and LOPE, and therefore to pinpoint the liable cell population for the disease.^34^ The overall maternal‐foetal interface placental cells can be clustered into 15 subpopulations, including EVT, villous cytotrophoblast (VCT), syncytiotrophoblast (SCT), endothelial cell, Hofbauer cell (HB), fibroblast, epithelial cell and macrophage (Figure 2B, left panel). Cell‐specific genes were identified for each subpopulation (examples shown in Figure 2B, right panel). We then calculated the intersections of these marker genes and the DEGs identified in EOPE and LOPE to determine the cell‐specificity of DEGs expression. A total of 94 upregulated and 206 downregulated DEGs were cell‐type‐specifically expressed in EOPE, while with only 16 and 6 corresponding DEGs found in LOPE (Figure 2C and Table S2). Therefore, we focused on EOPE for further analyses. As expected, the upregulated DEGs of EOPE were highly enriched in EVT (Figure 2D, left panel), which confirmed the role of EVT in EOPE pathogenesis. GO analysis of upregulated DEGs further demonstrated the dysfunction of HIF‐1α and angiogenesis signalling pathways in EVT (Figure 2E, top panel). Intriguingly, most of the downregulated DEGs were specifically expressed in placental fibroblast (Figure 2D, right panel). GO analysis of DEGs derived from fibroblast reminded that fibroblast was involved in angiogenesis and tissue morphogenesis as well (Figure 2E, bottom panel). Notably, we observed that angiotensin II Receptor Type 1 (*AGTR1*), the key receptor for angiotensin II, was highly expressed in placental fibroblast (Figure 2F). The elevated circulating angiotensin II type I receptor agonistic autoantibody (AT1‐AA) has been implicated in the pathogenesis of pre‐eclampsia by super‐activating AT1 receptor signalling in endothelium.^35^ The fibroblastic‐expression of *AGTR1* suggested that fibroblast also play roles in the renin/angiotensin signalling during pre‐eclampsia. Moreover, abnormal activation of fibroblasts has been reported in association with the fibrosis in pre‐eclamptic placentae by activating transforming growth factor β1 (TGFB1) signalling pathway.^36^ Our data showed that *TGFB1* was mainly expressed in the EVT, yet TGFB1‐activated fibrosis‐related factors, such as cellular communication network factor 2 (*CCN2*, also known as *CTGF*) and fibronectin1 (*FN1*), were highly expressed in both EVT and fibroblast (Figure 2F). Therefore, the fibrosis of pre‐eclamptic placenta might be a consequence of the interaction between EVT and fibroblast.

### 3.3 Comparative transcriptome profiling of maternal peripheral blood from EOPE and LOPE patients

**FIGURE 3:**
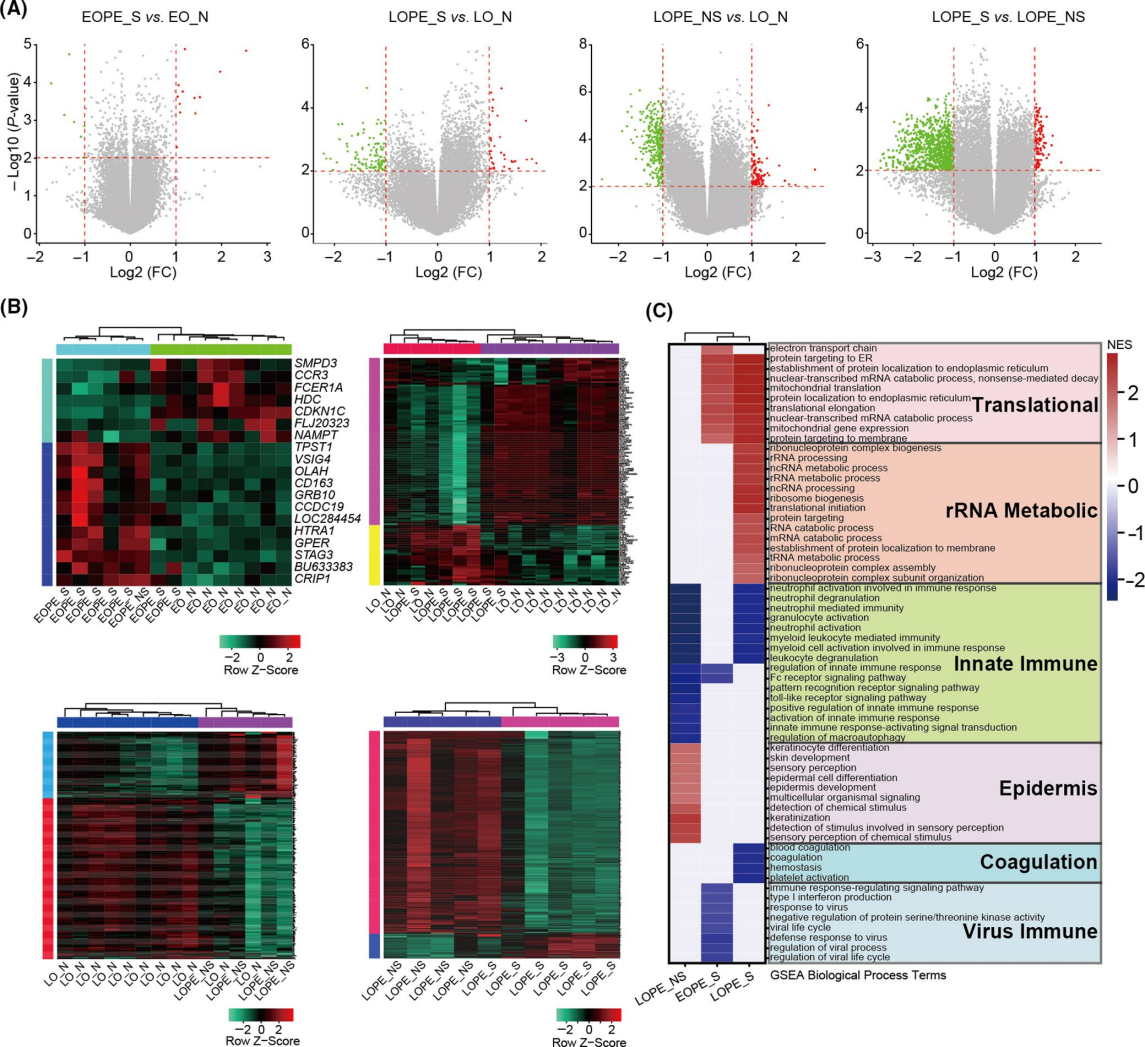
Transcription profiling analysis of maternal peripheral blood with EOPE and LOPE. A, Volcano plots showing DEGs in severe EOPE (n = 7), severe LOPE (n = 6), non‐severe LOPE (n = 4) and severe LOPE *vs*. non‐severe LOPE. The red, green, and grey dots indicate DEGs that considered to be upregulated, downregulated and non‐difference, respectively. Thresholds are indicated with dash lines. B, Heatmap presenting relative expression of DEGs in severe EOPE (top‐left), severe LOPE (top‐right), non‐severe LOPE (bottom‐left), and severe LOPE *vs*. non‐severe LOPE (bottom‐right). C, The significant GSEA biological process terms in each pre‐eclampsia group. EOPE_S, severe EOPE; EOPE_NS, non‐severe EOPE; EO_N, the normal group corresponding to EOPE; FC, fold change; LOPE_S, severe LOPE; LO_N, the normal group corresponding to LOPE and LOPE_NS, non‐severe LOPE

High similarity of placental transcriptomes between LOPE and healthy controls suggests that dysfunction of placenta is not the leading cause of LOPE. To this end, we considered the possibility to explore the pathogenesis of LOPE from maternal peripheral blood cells. We compared the maternal peripheral blood transcriptomes between different forms of pre‐eclampsia and their corresponding controls by deeply re‐analysing a public dataset.^17^ A total of 36 women with 18 patients and 18 healthy controls were included in this study. The 18 patients with pre‐eclampsia were further classified into four groups: severe EOPE, non‐severe EOPE (only one patient), severe LOPE and non‐severe LOPE, based on the clinical metadata provided in the original paper.^17^ The DEGs were identified between groups and their controls after data quality control and normalization (Figure 3A, Figure S4A‐B and Table S3). As expected, the number of DEGs in the EOPE was small, while both severe and non‐severe LOPE had obvious differences in gene expression profiles versus their respective controls, indicating the gene expression changes in maternal peripheral blood are more associated with LOPE (Figure 3A‐B). Sample correlation analysis confirmed the difference of maternal blood transcriptomes between EOPE and LOPE (Figure S4C), which was consistent with the placental results. Notably, gene expression profiles between severe and non‐severe LOPE were also obviously different, implying that the pathology of LOPE is heterogeneous depending on the severity or developmental stages of the disease (Figure 3A‐B and Figure S4C).

To investigate underlying biological processes of the diseases, GSEA was performed to distinguish altered pathways in EOPE and LOPE peripheral blood. Among all pathways, innate immune was commonly enriched in both severe and non‐severe LOPE, suggesting that the innate immune dysfunction can be one of the leading causes of LOPE (Figure 3C). Upregulation of innate immune response occurs during normal pregnancy, but its excessive activity is involved in the pathology of pre‐eclampsia.^37^ Our results showed that neutrophil mediated immunity was strongly associated with LOPE (Figure 3C). A pathologic explanation is that maternal inflammatory response causes neutrophil activation, leading to the release of cytokines such as calprotectin into circulation which in turn induces the maternal LOPE symptoms.^38^ On the other hand, a large number of biologic processes were differentially enriched in severe and non‐severe LOPE. For example, the changes of epidermis associated biological processes were prominent in non‐severe LOPE, while coagulation, endoplasmic reticulum (ER) and mitochondrial translation and ribosomal RNA (rRNA) metabolism were strongly associated with severe LOPE (Figure 3C). It is known that ER translation and rRNA metabolic process are critical for protein synthesis, folding and trafficking, which are often regarded as conduits to human disease.^39^, ^40^

**FIGURE 4:**
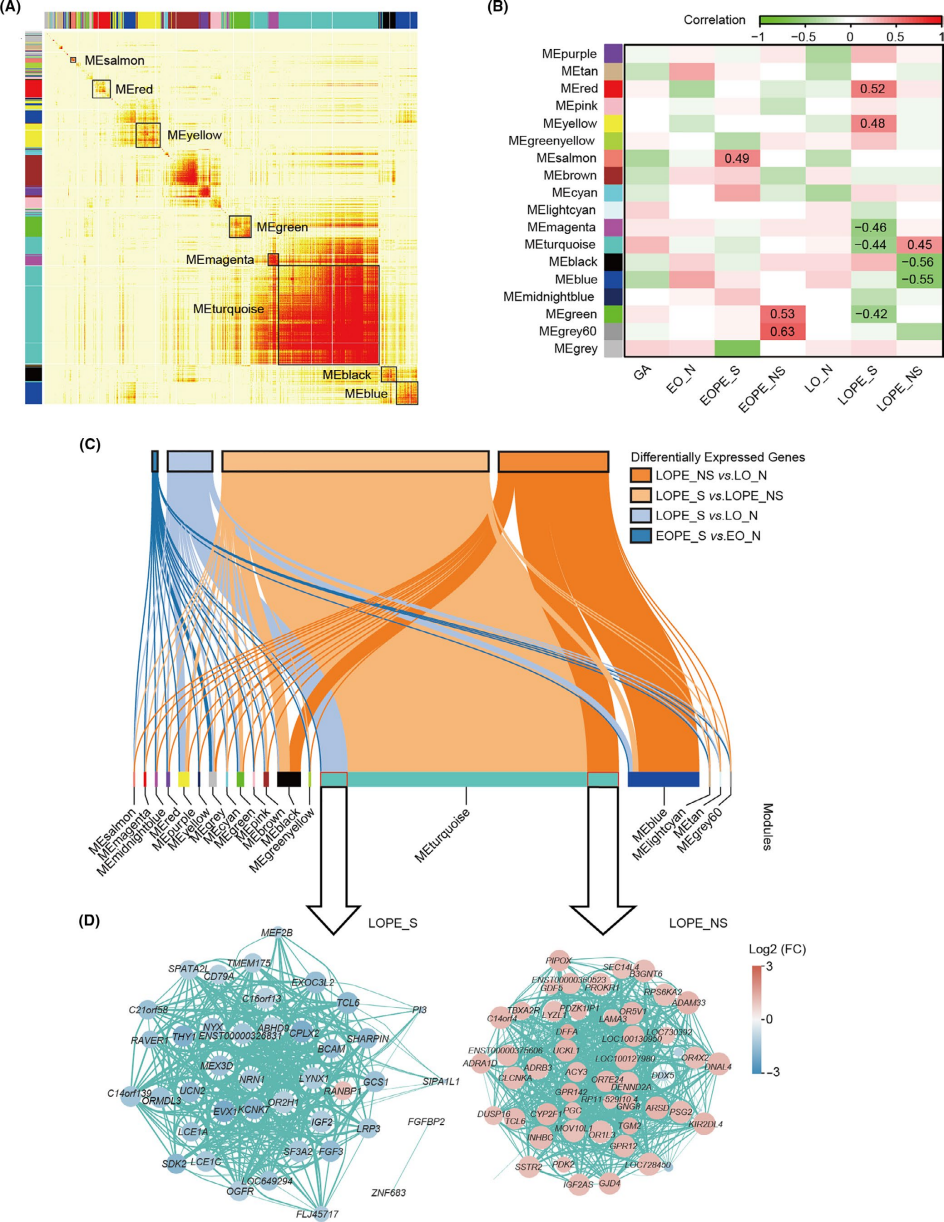
Co‐expression network analysis of DEGs in peripheral blood. A, Heatmap presenting the 18 clusters of HVGs. The colours of horizontal axis and vertical axis represent different modules. The typical modules are noted in the map with squares. B, Heatmap presenting the correlation between different gene modules and disease conditions. C, Visualization of the overlap between DEGs in modules and disease conditions. D, Visualization of co‐expression network and hub DEGs of severe LOPE in MEturquoise module (left panel) and non‐severe LOPE in MEturquoise module (right panel). Red and blue filled nodes represent positively and negatively regulated genes, respectively. Node size is correlated with the degree of the connectivity of the genes. Colour of lines indicates the colour code of the module. HVGs, highly variable genes; EOPE_S, severe EOPE; EOPE_NS, non‐severe EOPE; EO_N, the normal group corresponding to EOPE; LOPE_S, severe LOPE; LO_N, the normal group corresponding to LOPE and LOPE_NS, non‐severe LOPE

To specify pivotal regulators in the different pathology of severe and non‐severe LOPE, a co‐expression network analysis was performed. 18 gene modules (labelled with colours, such as MEturquoise) were generated through calculating the correlation of the HVGs screened from maternal peripheral blood transcriptomes (Figure 4A). We then analysed the relationships between these gene modules and status of pre‐eclamptic diseases. As shown in the heatmap of module‐diseases correlations, 5 modules were in tight connection with severe LOPE, with another 3 showing a more enriched tendency with non‐severe LOPE (Figure 4B). Interestingly, genes in MEturquoise exhibited closer but inverse correlation output with both severe and non‐severe LOPE (Figure 4B). To uncover the potential involvement, we explored the genes that were responsible for this divergence and their regulatory networks. Through examining the intersection nodes between genes in each module and DEGs, we found that part of these genes in MEturquoise were upregulated in non‐severe LOPE, while a portion of the rest were downregulated in severe LOPE (Figure 4C‐D). These different genes with opposite expression patterns in the same module strongly suggest that LOPE progresses differentially depending on the developmental stage or severity of the disease. Additionally, nearly all genes differentially expressed between severe LOPE and non‐severe LOPE were enriched in MEturquoise module, indicating that these DGEs were pivotal in determining the severity of LOPE (Figure 4C).

### 3.4 Identification of novel biomarkers for EOPE and LOPE diagnosis

**FIGURE 5:**
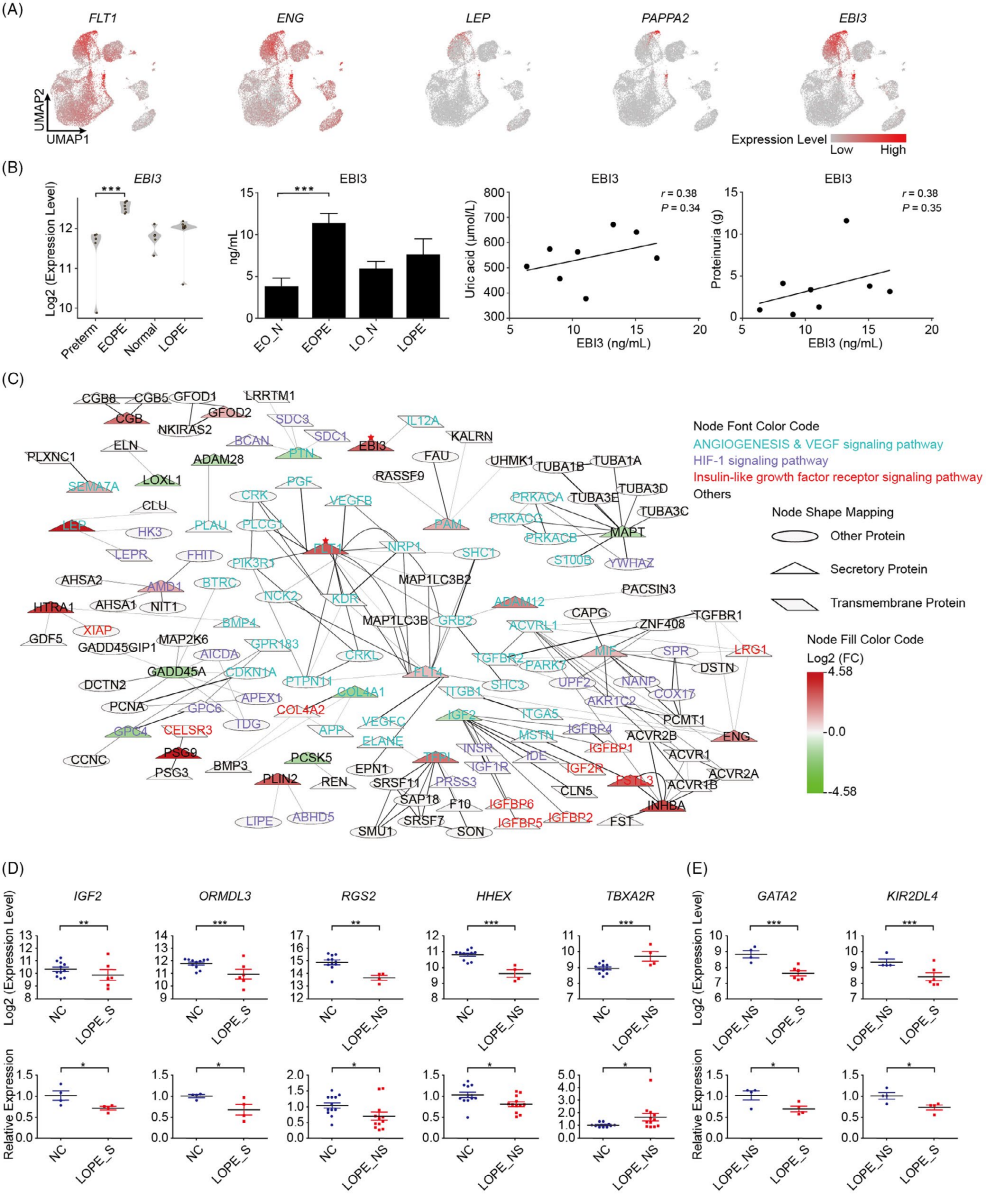
Potential biomarkers for EOPE and LOPE. A, UMAP plots showing expression levels of known and novel biomarkers in the atlas of maternal‐foetal interface. B, Plasma EBI3 levels were elevated in EOPE (n = 8) but not LOPE (n = 6) and presented positively correlation tendency to uric acid and proteinuria. The bioinformatic, experimental verified EBI3 expression levels and the correlation to uric acid and proteinuria were presented from left to right, respectively. C, A network of secretory proteins and their targets. The colour of the gene name indicates the signalling pathways to which it belongs; the node shape indicates the cellular location of the protein; the filled colour of each node indicates the expression change of the gene in EOPE patients; the red star indicates the proteins discussed in the main text. D, Scatter plots showing the bioinformatic (top panel) and qPCR verified (bottom panel) expression levels of newly identified biomarkers for LOPE (For experimental data, LOPE_S, n = 4; NC for LOPE_S, n=4; LOPE_NS, n = 12; NC for LOPE_NS, n=12). E, Scatter plots showing the bioinformatic (top panel) and qPCR verified (bottom panel) expression levels of newly identified biomarkers that could be used to distinguish the severity of LOPE (For experimental data, severe and non‐severe LOPE, n = 4). Data are presented as mean ± SEM and compared by the empirical Bayes moderated t‐statistics test (for bioinformatic data) and Student's t‐test (for experimental data). *P < .05, **P < .01, ***P < .001. EO_N, the normal group corresponding to EOPE; FC, fold change; LOPE_S, severe LOPE; LO_N, the normal group corresponding to LOPE and LOPE_NS, non‐severe LOPE

Given the low efficiency and sensitivity of the current biomarkers and their limited application in LOPE diagnosis, we then aimed to explore novel clinical biomarkers especially for those can be noninvasively detected from maternal peripheral blood.^11^ The potential biomarkers are most likely composed of secretory proteins either produced by maternal‐foetal interface tissues or generated directly from circulating maternal blood. Thus, we firstly explored secretory proteins from DEGs which were specifically expressed in EVT or SCT (maternal‐foetal interface cell types) (see Figure 2D). Importantly, a large proportion of the DEGs elevated in EOPE were secretory proteins, with a total of 31 and 4 factors were identified in EVT and SCT, respectively (Table 1). We found that the well‐accepted diagnostic markers, *FLT1*, *LEP*, *ENG* and *PAPPA2*, were highly expressed in EVT and SCT, which revealed the origin cell types of these key pathogenesis factors in EOPE (Figure 5A). Most importantly, a large number of novel potential biomarkers were captured, such as the upregulated genes perilipin 2 (*PLIN2*), fms related receptor tyrosine kinase 4 (*FLT4*), epstein‐barr virus induced 3 (*EBI3*) as well as the downregulated genes proprotein convertase subtilisin/kexin type 5 (*PCSK5*), glypican 4 (*GPC4*), lysyl oxidase like 1 (*LOXL1*) (Table 1). Among the upregulated factors, EBI3, the subunit of immune‐regulatory cytokines, generally increases during normal pregnancy in maternal plasma.^41^ Our results clearly showed that *EBI3* was elevated in its EVT‐origin of EOPE, suggesting that detecting excessive *EBI3* from maternal plasma has great potential for the diagnosis of EOPE (Figure 5A). To verify this hypothesis, we examined the circulating levels of EBI3 in EOPE and LOPE patients. While most of the blood parameters were normal in EOPE patients (Table 2), indeed, we found that the circulating EBI3 was increased over two times (Figure 5B and Table 2), which was not observed in LOPE. Besides, the combination of clinic parameters showed trends of positively correlation of plasma EBI3 levels to uric acid and proteinuria, strongly indicating that the elevated EBI3 can be a very sensitive biomarker of EOPE (Figure 5B).

**TABLE 1:** The list of secretory proteins derived from EVT and SCT

Gene name	Log2 (FC)	*P*‐value	Cell type	Gene name	Log2 (FC)	*P*‐value	Cell type
*ADAM12*	2.14	***	EVT	*PAPPA2*	4.77	***	EVT
*AMD1*	1.31	***	EVT	*PLIN2*	2.86	***	EVT
*CGB*	2.52	***	EVT	*PRSS8*	1.01	**	EVT
*EBI3*	2.77	***	EVT	*PSG9*	4.58	***	SCT
*ENG*	2.03	***	EVT	*SEMA7A*	1.33	***	EVT
*FLT1*	1.95	***	EVT	*TFPI*	2.43	**	EVT
*FLT4*	1.02	**	EVT	*ADAMTS1*	‐1.08	***	EVT
*FSTL3*	2.94	***	EVT	*ADAM28*	‐1.07	***	EVT
*GFOD2*	1.26	***	EVT	*COL4A1*	‐1.65	***	EVT
*GREM2*	2.40	**	SCT	*GADD45A*	‐1.39	***	EVT
*HILPDA*	1.64	***	EVT	*GPC1*	‐1.01	***	EVT
*HTRA1*	3.60	***	EVT	*GPC4*	‐1.68	***	EVT
*HTRA4*	3.34	**	EVT	*IGF2*	‐1.03	**	EVT
*INHBA*	3.31	***	SCT	*LOXL1*	‐1.31	***	EVT
*LEP*	4.33	***	EVT	*MAPT*	‐1.18	***	EVT
*MIF*	1.13	***	EVT	*PCSK5*	‐1.35	***	EVT
*NPB*	1.63	**	SCT	*PTN*	‐1.21	**	EVT
*PAM*	1.27	**	EVT				

Note

Data are compared by the empirical Bayes moderated t‐statistics test. *P < .05, **P < .01, ***P < .001.

Abbreviations: EVT, extravillous trophoblast; FC, fold change and SCT, syncytiotrophoblast.

**TABLE 2:** Clinical characteristics of healthy pregnant women and women with pre‐eclampsia

	EO_N (n = 7)	LO_N (n = 13)	EOPE (n = 8)	LOPE_S (n = 4)	LOPE_NS (n = 12)
Age, y	32.0 ± 3.4	30.7 ± 3.9	34.5 ± 6.9	32.3 ± 5.4	32.3 ± 5.0
Gestational age at sampling, wk	29.7 ± 2.7	37.1 ± 1.4	30.3 ± 2.9	36.9 ± 1.0	37.3 ± 1.3
Systolic blood pressure, mmHg	110.0 ± 7.3	106.4 ± 9.9	160.1 ± 23.3*	155.8 ± 15.3*	124.0 ± 13.2*
Diastolic blood pressure, mmHg	66.6 ± 12.2	61.2 ± 17.0	105 ± 14.7*	99.8 ± 15.9*	79.8 ± 12.3*
Urinary protein, g	Negative	Negative	3.6 ± 3.2	5.3 ± 5.5	0.9 ± 1.3
Uric acid, μmol/L	354.2 ± 137.1^†^	394.4 ± 153.2	541.2 ± 89.4*	586.0 ± 170.5	388.5 ± 104.6^‡^
Creatinine, μmol/L	40.7 ± 5.0	47.4 ± 10.1	63 ± 15.5*	63.3 ± 18.7	52.2 ± 10.8
Thrombocyte, 10^9/L	241.3 ± 61.6	200.5 ± 45.4	219.1 ± 62.4	187.3 ± 39.9	205.0 ± 53.4
haematocrit value, %	33.3 ± 2.0	33.9 ± 4.5	34.8 ± 2.5	35.2 ± 4.1	33.2 ± 3.2
Alanine transaminase, U/L	14.1 ± 10.4	11.5 ± 5.9	19 ± 10.4	24.3 ± 14.4*	14.3 ± 5.5
Aspartate transaminase, U/L	17.4 ± 3.8	17.8 ± 4.2	20.3 ± 4.4	31.5 ± 13.6*	21.7 ± 8.6
EBI3, ng/mL	3.7 ± 2.6	5.8 ± 2.2 (n = 6)	11.3 ± 3.3*	7.5 ± 4.4 (n = 6)	

Note

Data are presented as mean ± SD. EO_N, the normal group corresponding to EOPE; LOPE_S, severe LOPE; LO_N, the normal group corresponding to LOPE and LOPE_NS, non‐severe LOPE.

All statistical significance was calculated by Student's t‐test.

* Vs the corresponding normal group. *P* < .05.

^†^ n = 6, there is one healthy pregnant woman who did not have the result of uric acid.

^‡^ Vs the women with severe LOPE *P* < .05.

To exclude the potential interference induced by the factors derived from maternal haematopoietic system, the expression patterns of the above factors were then tested in an integrated single‐cell map which contained placental and matched blood mononuclear cells transcriptomic profiles (Figure S5A).^34^ We found that both the classical diagnostic markers (such as *FLT1*, *ENG*, *LEP* and *PAPPA2*), and novel biomarkers (such as *EBI3*, *FLT4*, *LOXL1* and *GPC4*) showed highly specific expressions in placenta but not in blood (Figure S5B‐C).

To unveil the detailed biological and pathological functions of these secretory factors, protein‐protein interactions were further analysed. These interacting proteins were mainly involved in angiogenesis, vascular endothelial growth factor (VEGF), HIF‐1 and insulin‐like growth factor receptor signalling pathways (Figure 5C). As expected, FLT1 was at the centre of angiogenesis signalling pathways (Figure 5C). It has been reported that the secreted form of FLT1, sFLT1, exerts antiangiogenic effects through binding to proangiogenic proteins VEGF and placental growth factor (PGF) to inhibit their function.^6^ In our data, more interactions between *FLT1* and its targets were disclosed, suggesting that *FLT1* may facilitate pre‐eclamptic symptoms through other unrevealed mechanisms. Moreover, we identified glycoprotein neuropilin 1 (*NRP1*) as a target of *FLT1*. *NRP1* had been reported to be associated with foetal growth restriction, a clinical implication of pre‐eclampsia, although the mechanism was largely unknown.^42^

For those potential biomarkers produced directly from maternal peripheral blood, we searched for the hub genes in each network composed of the overlap between DEGs and gene regulatory modules of either severe (Figure S6A) or non‐severe LOPE (Figure S6B). Importantly, some of the hub genes in the networks including insulin‐like growth factor 2 (*IGF2*) and regulator of G protein signalling 2 (*RGS2*) which play key roles in the progress of pre‐eclampsia were reported downregulated in pre‐eclamptic placenta.^43^, ^44^ Our bioinformatic results further showed that expression levels of these genes were reduced in LOPE and might be the key regulators of LOPE pathology. We therefore considered the possibility to use these hub genes as biomarkers of LOPE (Table S4). Indeed, the mRNA levels of *IGF2* and ORMDL Sphingolipid Biosynthesis Regulator 3 (*ORMDL3*) were extremely decreased in maternal blood of severe LOPE patients (Figure 5D and metadata see Table 2). Similarly, the *RGS2* and haematopoietically expressed homeobox (*HHEX*) were accordingly downregulated, and thromboxane A2 receptor (*TBXA2R*) was upregulated in non‐severe LOPE patients (Figure 5D). Importantly, the DEGs between severe *vs*. non‐severe LOPE were also able to be used to distinguish the severity of LOPE with experimentally verification of GATA binding protein 2 (*GATA2*) and killer cell immunoglobulin like receptor, two Ig domains and long cytoplasmic tail 4 (*KIR2DL4*) (Figure 5E). Therefore, these maternal blood‐derived factors present as favourable diagnostic biomarkers and potential therapeutic targets for LOPE.

## 4 DISCUSSION

Although relevant symptoms have been documented for two centuries, the pathogenesis of pre‐eclampsia remains poorly understood, limiting effective treatment. One main reason is that the highly variable clinical features of pre‐eclampsia even complicate a clear definition and classification of the disease. Until recently, pre‐eclampsia has been defined by the International Society for the Study of Hypertension in Pregnancy (ISSHP) as new‐onset hypertension accompanied by one or more other features: proteinuria, other maternal organ dysfunction, or haematological involvement, and/or uteroplacental dysfunction.^45^ It is also generally accepted that there are two varieties of pre‐eclampsia, that is, EOPE and LOPE. Knowing that EOPE and LOPE are in general placental‐ and maternal‐ origin diseases, however, the detailed pathophysiology underlying has never been systemically investigated and compared. We integrated the resources of transcriptomic data from placentae and maternal peripheral blood, and performed comprehensive analyses to compare EOPE, LOPE, and their relevant controls. We identified DEGs from placentae and peripheral blood transcriptomes of EOPE and LOPE patients. By comparing DEGs, we found fundamental pathologic differences between EOPE and LOPE. Our data also provide conclusive evidence, in both placentae and peripheral blood, that distinct genes and signalling pathways are involved in the EOPE and LOPE diseases, respectively. Hence, we proposed that the EOPE and LOPE definitely should be treated as two diseases entities.

The difficulty in mechanistic investigation of pre‐eclampsia is at least partly due to the lack of golden biomarkers. Recently, anti‐/angiogenic factors have emerged as important indicators of pre‐eclampsia. The placental levels of *FLT1*, *PGF* and *ENG* differ significantly between patients with pre‐eclampsia and those of normotensive gravidas. The ratio of sFLT1/sENG to PGF, as well as the concentration of metabolic and inflammatory factors, such as LEP and PAPPA2, has also been reported to elevate in the plasma of pre‐eclampsia patients.^8^, ^9^, ^10^ Despite it is known that they are likely originated from defective placenta, the culpable cell type is yet not elucidated as it is technically not realistic to isolate all types of cells and check their transcriptome/proteome separately. The recent advances of single‐cell RNA sequencing (scRNAseq) provides opportunities for tracking the differentially expressing genes with extremely high resolution and in very complicated systems including maternal‐foetal interface.^34^ We took this advantage and mapped all DEGs in EOPE and LOPE placentae into a single‐cell atlas of maternal‐foetal interface. For the first time, we provide conclusive evidence that the upregulated and downregulated genes in EOPE are mainly derived from EVT and foetal fibroblast, respectively. Importantly, we found that the traditional biomarkers, such as *FLT1*, *ENG*, *LEP* and *PAPPA2* were only upregulated in the EOPE, but not LOPE placenta. A special caution therefore should be taken in using these markers in the ‘overall’ pre‐eclampsia diagnosis or prediction. Consistent with this idea, results of recent nonintervention cohort studies indicated that both PGF and sFLT1 presented favourable potential for the prediction of EOPE, while screening for LOPE yielded a much poorer performance.^46^, ^47^

The mapping of EOPE DEGs to the cell atlas of maternal‐foetal interface also allowed us to identify many novel factors derived from EVT and SCT (Table 1). The direct contact of EVT and SCT with maternal blood in uterine spiral arteries allows those placenta‐secreted proteins release to maternal circulation and thereby could be detected from peripheral blood (Figure 2A). A comparative and network analysis of DEGs in LOPE blood cell transcriptomes also allowed us to identify several potential biomarkers and therapeutic targets for LOPE. Most importantly, we experimentally validated the credibility of our method using both patient plasma and peripheral blood cells. We therefore provide an approach to precisely identify maternal blood detectable biomarkers for placental‐origin disease, which might be utilized in a wild spectrum of ‘disorders of placentation syndromes’, including spontaneous miscarriage, abruptio placentae, foetal growth restriction and premature delivery.

Our work also unveils several new pathological insights into both EOPE and LOPE. For instance, we found a large number of genes were downregulated in the foetal fibroblast in EOPE (Figure 2D). It is known that fibrosis is an important factor to cause pre‐eclampsia, as we discussed above. However, the role and detailed regulatory mechanism has not been well‐documented, which still requires deeper investigation. Besides, we identified several signalling pathways and regulators, such as *IGF2* and *ORMDL3 vs. RGS2*, *HHEX*, *TBXA2R* that regulate severe and non‐severe LOPE, respectively. It is of interest to further explore the pathogenesis of LOPE at different severity and disease developmental stages.

In summary, our work provides strong evidence showing the distinct pathology of EOPE and LOPE. While the EOPE has abnormal function of EVT and fibroblasts in the maternal‐foetal interface, LOPE shows gene expression alteration in peripheral blood. We suggest that the previously considered two forms of pre‐eclampsia should be treated as two disease entities. Based on the difference in pathology, the novel biomarkers, either secretory factors or blood cell transcripts, were identified for both EOPE and LOPE, which open new venues for developing new diagnostic and therapeutic strategies for both diseases.

## CONFLICT OF INTEREST

The authors declare that they have no competing interest.

## AUTHOR CONTRIBUTIONS

SW and HZ conceived and designed the project. FG, BZ, HY analysed the data and performed the experiments with the help from YF, YW, JH, MC, XL, ZS, LL, PH. FG, BZ, HY, SW and HZ wrote the manuscript with the contributions from YF, YW and APX. All authors read and approved the final manuscript.

## Supporting information

Fig S1

Fig S2

Fig S3

Fig S4

Fig S5

Fig S6

Table S1

Table S2

Table S3

Table S4

Table S5

## Data Availability

The data that support the findings of this study are available from the corresponding authors on reasonable request.
