# Supplementary figures and images for: Systemic transcriptome comparison between early‐ And late‐onset pre‐eclampsia shows distinct pathology and novel biomarkers
Source: Cell Prolif. 2020 Dec 17;54(2):e12968. doi: 10.1111/cpr.12968 (PMC7848957; doi:10.1111/cpr.12968)

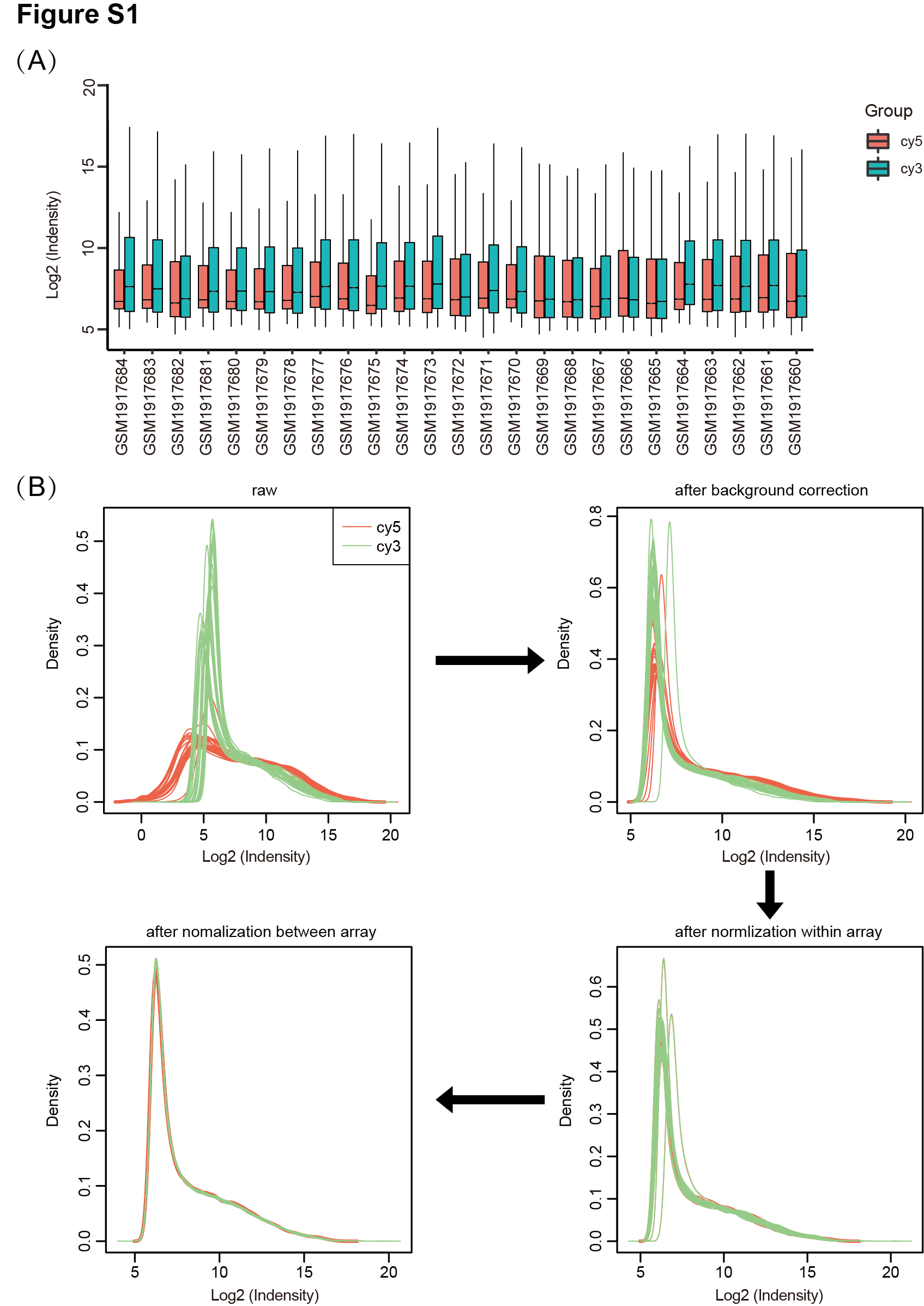

Supplement: Supplementary file 1 — Fig S1 [file CPR-54-e12968-s001.png]

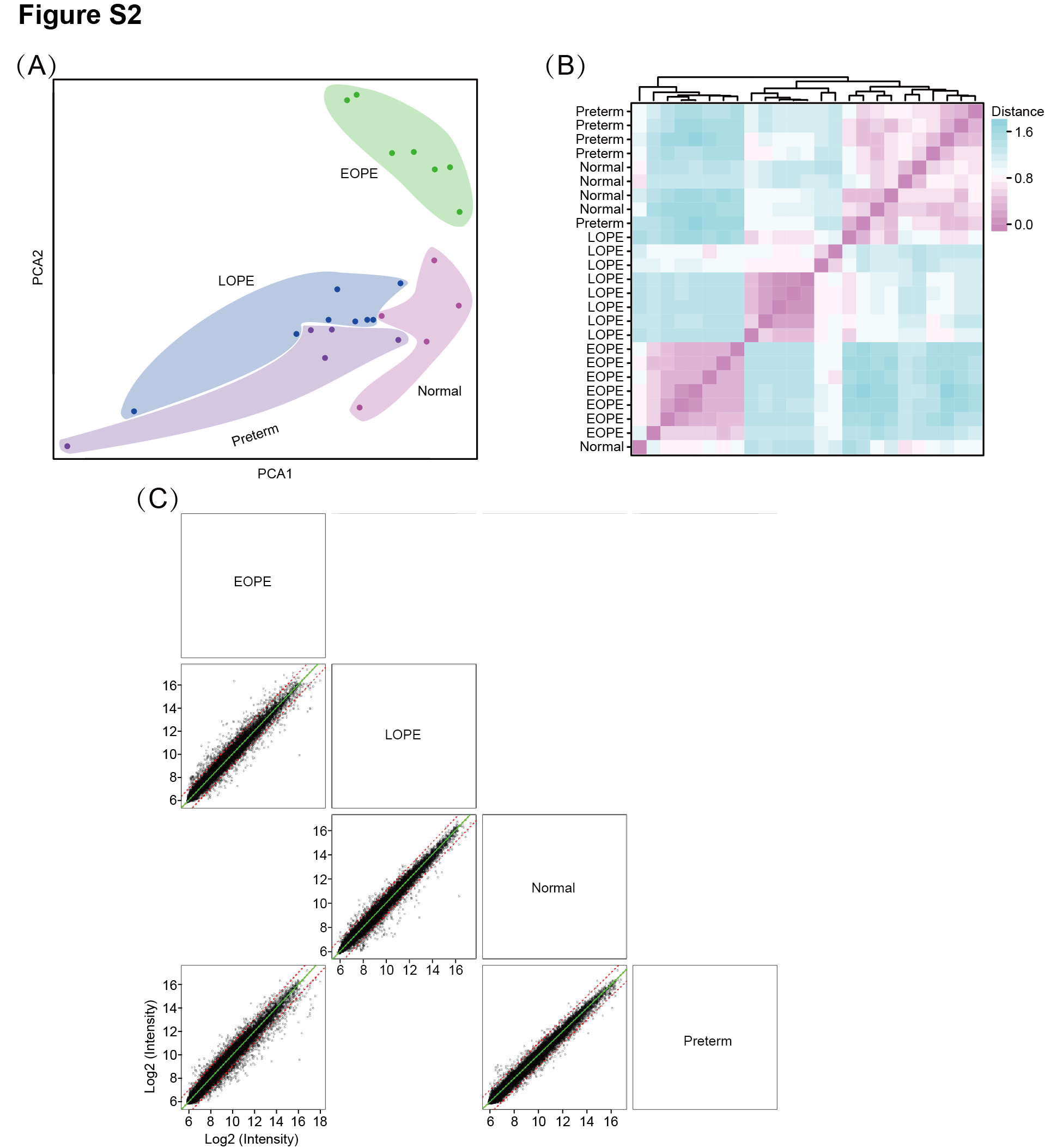

Supplement: Supplementary file 2 — Fig S2 [file CPR-54-e12968-s002.png]

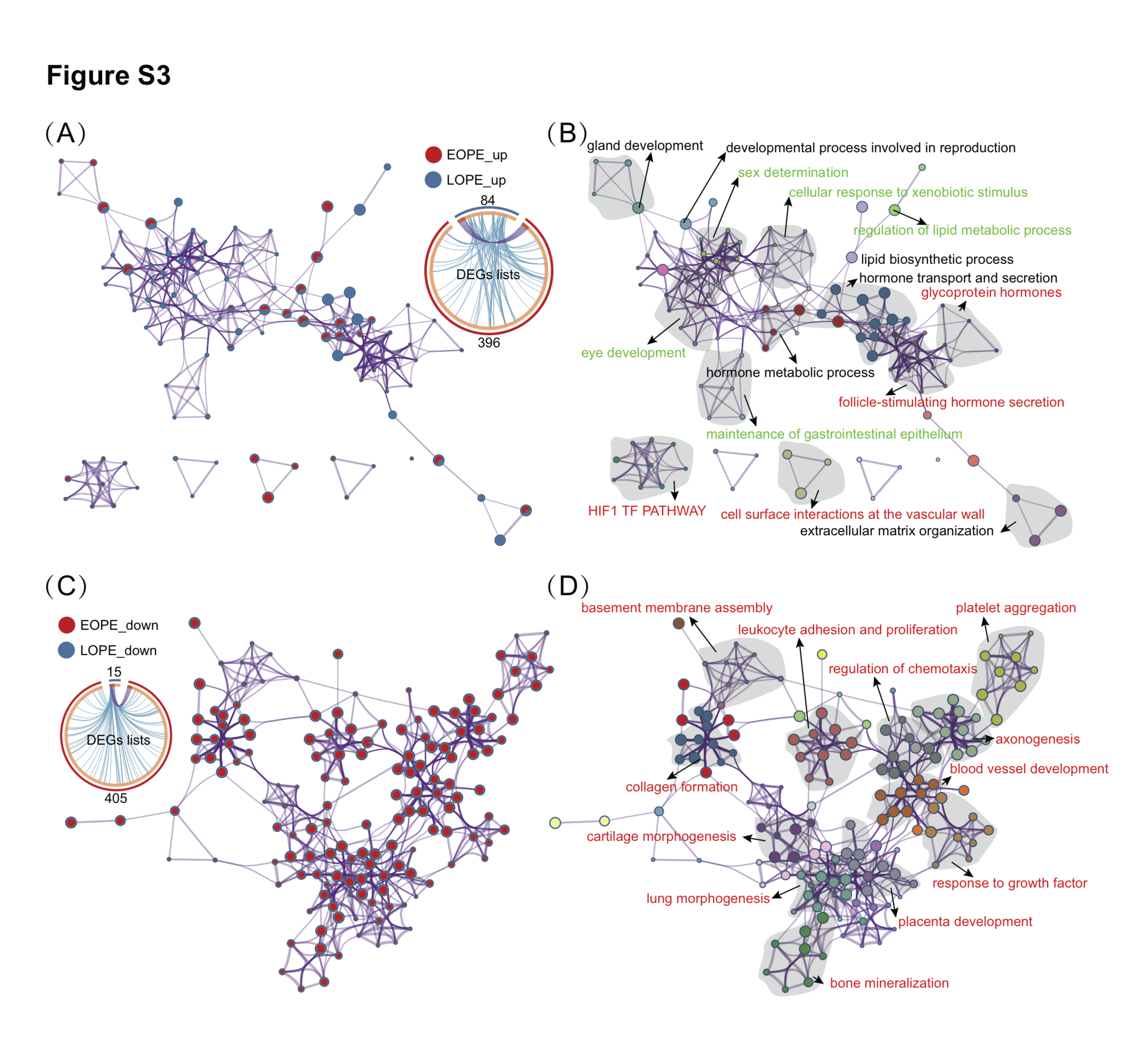

Supplement: Supplementary file 3 — Fig S3 [file CPR-54-e12968-s003.png]

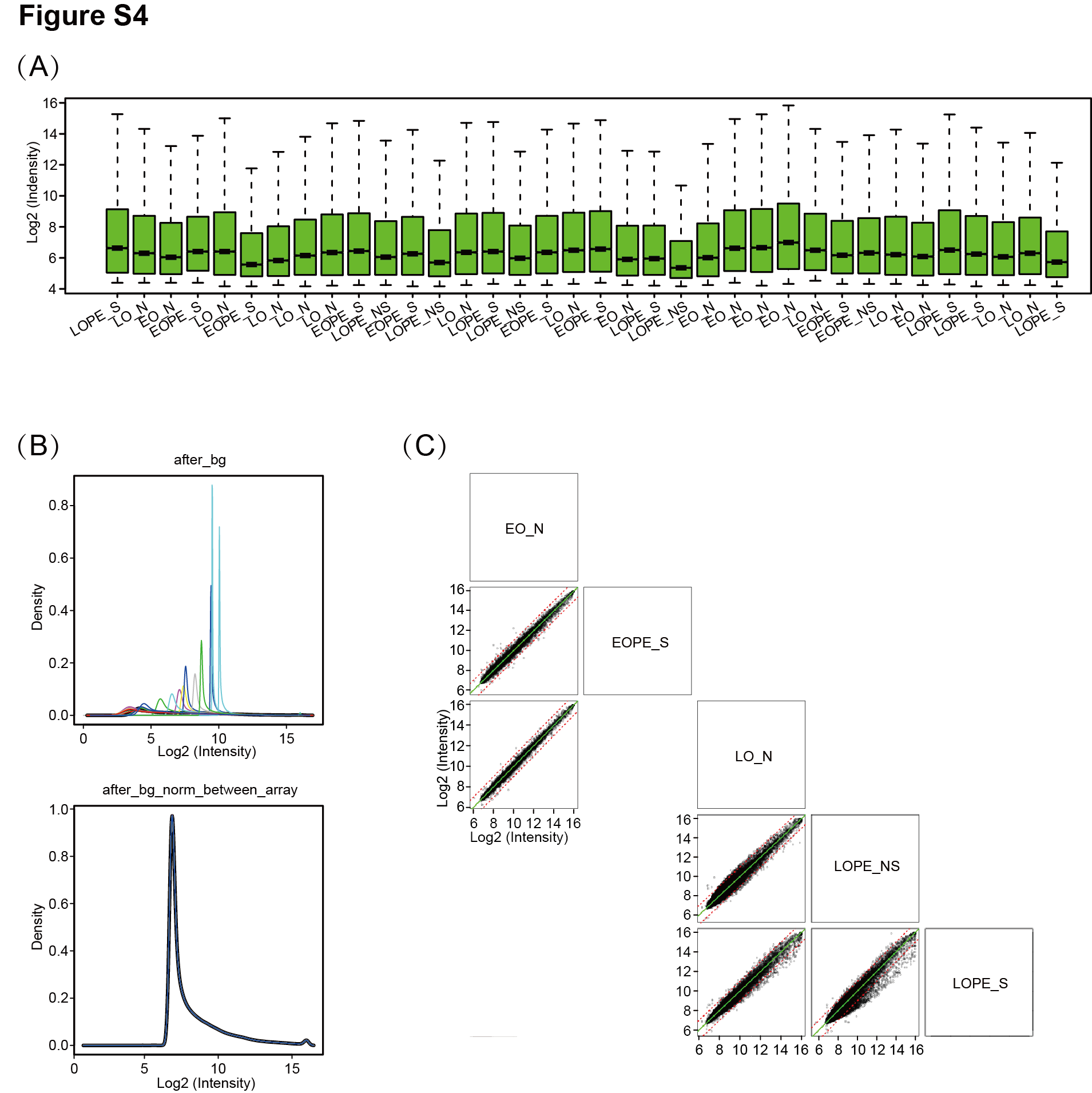

Supplement: Supplementary file 4 — Fig S4 [file CPR-54-e12968-s004.png]

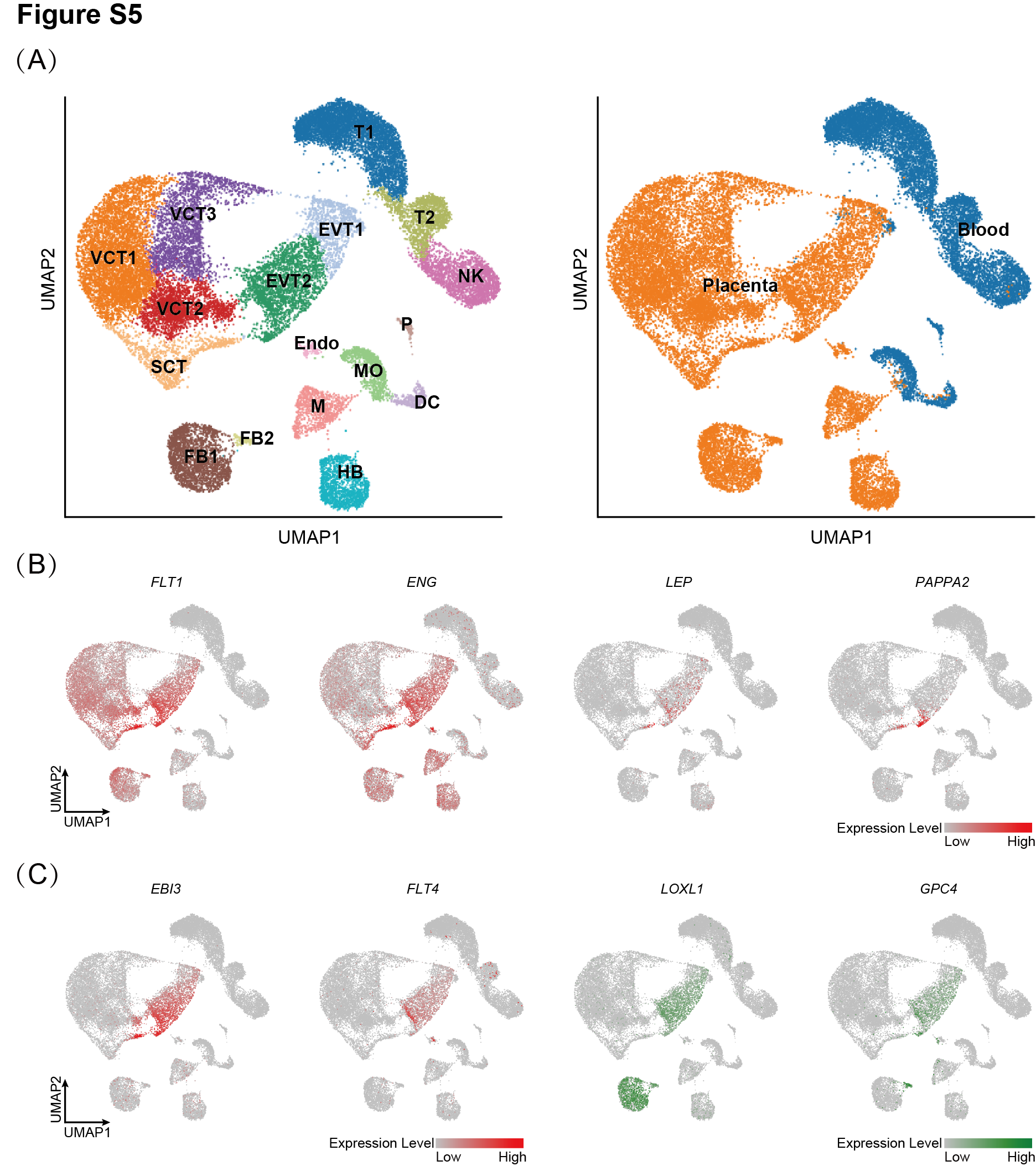

Supplement: Supplementary file 5 — Fig S5 [file CPR-54-e12968-s005.png]

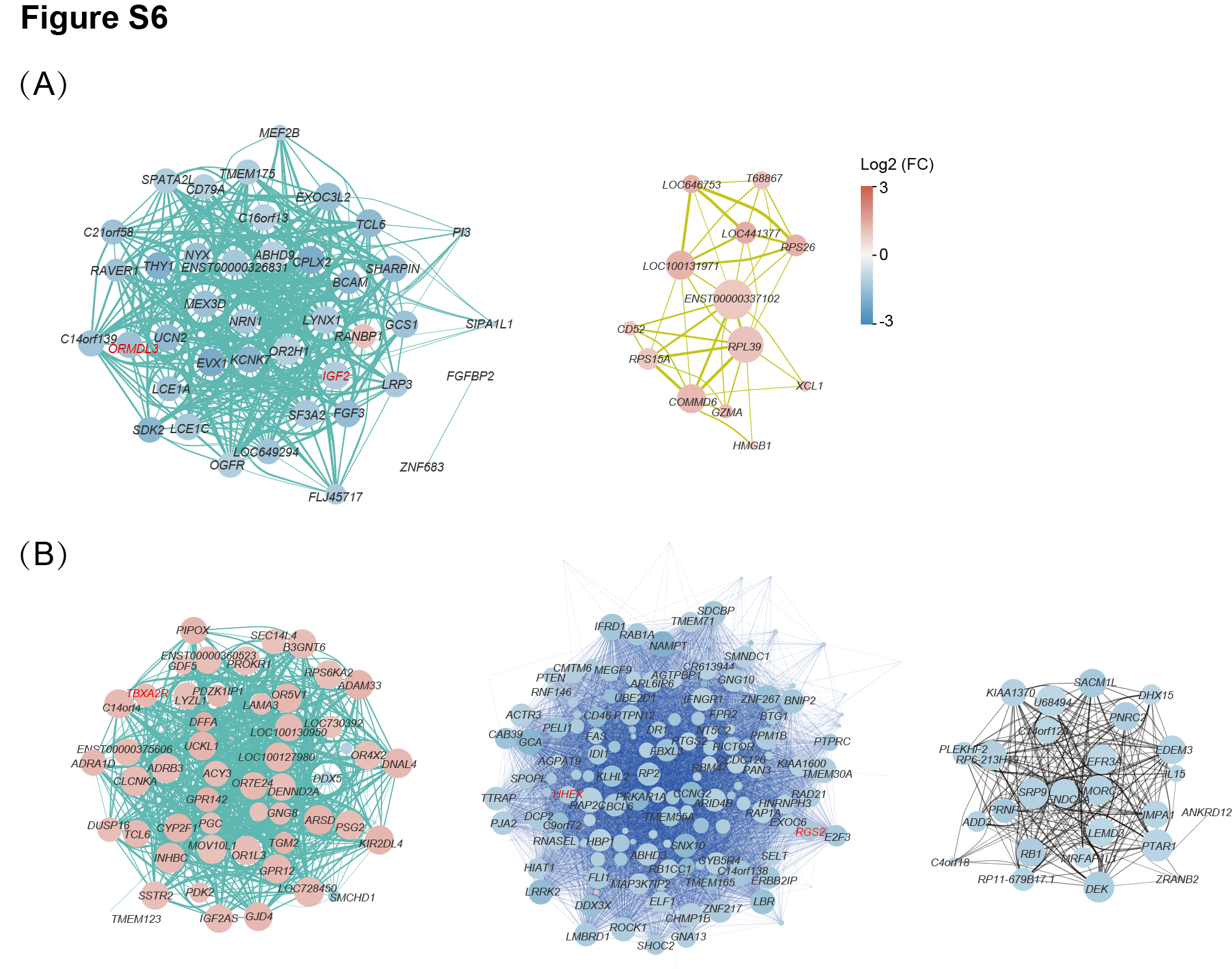

Supplement: Supplementary file 6 — Fig S6 [file CPR-54-e12968-s006.png]
