# Supplementary material for: Systemic transcriptome comparison between early‐ And late‐onset pre‐eclampsia shows distinct pathology and novel biomarkers
Source: Cell Prolif. 2020 Dec 17;54(2):e12968. doi: 10.1111/cpr.12968 (PMC7848957; doi:10.1111/cpr.12968)
Supplement: Supplementary file 11 — Table S5 [file CPR-54-e12968-s011.docx]

**TABLE S5 Primers for qPCR**

| Gene | Primer sequence (5’-3’) | |
| --- | --- | --- |
| *RGS2* | Forward | CAGAAGCATTTGACGAGCTG |
|  | Reverse | TCACAGGCCAGCCAGAAT |
| *HHEX* | Forward | GGCCTCTGCATAAAAGGAAAG |
|  | Reverse | TTTGACCTGTCTCTCGCTGA |
| *TBXA2R* | Forward | GGGACATAGAGGATTCGGTTC |
|  | Reverse | CCTGTAGCCCTCCCATTACA |
| *IGF2* | Forward | CTTCTCACCTTCTTGGCCTTC |
|  | Reverse | CGGAAACAGCACTCCTCAA |
| *ORMDL3* | Forward | TGACCATCACACCCATCG |
|  | Reverse | GCAGCTTGGGGATAAGCA |
| *GATA2* | Forward | CGAGCTAGGGGAGGGAAC |
|  | Reverse | GGTAGGAGCTGGGGGTAGAG |
| *KIR2DL4* | Forward | CTGCGGGACACAGAACAGT |
|  | Reverse | TGTGAAAATGCAGTGATCCAA |
| *RPL13A* | Forward | CTCAAGGTGTTTGACGGCATCC |
|  | Reverse | TACTTCCAGCCAACCTCGTGAG |
